# 

**Published:** 1890-01-18

**Authors:** 


### NOTICE TO CORRESPONDENTS.

All MS., Letters, Books for Review, and other matters intended for the Editor, should be addressed The Editor, The Lodge, Porchester Square, London, W.

### Notice to Advertisers and Subscribers.

All Advertisements, Orders for Copies of The Hospital, and Business Communications should be addressed to The Publisher, *not to the Editor*, The Hospital, 139-140, Salisbury-court, Fleet-street, E.C.

### Bound Volumes of "The Hospital."

Vol. VI., containing full page portraits of T.R.H. the Prince and Princess of Wales, and Miss Florence Nightingale, is now ready, 4s., post free. Cases for binding, may be had of the Publisher, at 1s. 6d. each.

### To Residents, Secretaries, and Matrons

The Editor will be glad to have the Regulations and each Report as issued by Asylums, Hospitals, Convalescent and Nursing Institutions, as well as those of other Charities, and an early copy in duplicate of all Appeals and Festival Notices, etc., addressed to *The Editor, The Lodge, Porchester-square, London, W.* Any superintendent, secretary, matron, or other chief official who regularly sends such information may be registered, on application to the Publisher, to receive free of cost a cover for binding The Hospital in half-yearly volumes.

### Scraps and Gleanings.

Polly Thompson, aged 103, is now living in Camberwell Workhouse.

Thames Ditton is to have a mortuary; it is not to cost more than £100.

Dr. Botkin, who lately died at Mentone, was Russia's most celebrated physician.

Dr. Lund, late of Newcastle, has been appointed house surgeon at Doncaster.

A boy, aged 14, died at St. George's Hospital last week from hydrophobia.

Prof. Westphal, of Berlin, a specialist on mental diseases, has himself become insane.

Wrexham working-men have determined to contribute 50 guineas annually to the infirmary.

Grantham Hospital ball was a very brilliant affair, all the county families being well represented.

Hospital Sunday in Aberdeen resulted in the collection of £704, compared with £685 collected last year.

Richmond Hospital has received over £400 from the collecting-boxes in the town during six months' collecting.

The Commissioners of the New York Quarantine Department are having a yacht built for their special use.

The Dewsbury Industrial Society has contributed £25 for a testimonial to the hon. medical staff of the infirmary.

Rotherham Hospital has admitted 85 in-patients during the last quarter. Mr. Pigott has been appointed dispenser.

Prince Albert Victor has extended his patronage to the Leper Asylum as a permanent memorial of his visit to India.

An alarming epidemic of measles is raging at Alton, Hants. Over 300 cases are reported, and several deaths have taken place.

Judge Bristowe has given £50 to Nottingham General Hospital, besides paying for all the attention he has received there.

Harrogate Cottage Hospital acknowledges £45 from the working-men of the town, an increase of £7 on last year's collection.

Mr. Edward Ecroyd, J.P., of Nelson, has given £500 for establishing a children's ward at the Victoria Hospital for Burnley and district.

The Heckmondwike Co-operative Society have voted £5 to the St. John's Ambulance Association, and £25 to the Dewsbury Infirmary.

"Dr." Rudolph Wieczorek, an old man of New York, was fined 50dols. in the Court of Special Sessions for practising medicine without a certificate.

The Penny Dinners' Committee in Croydon have renewed their operations at the various Board schools, and are serving some hundreds of penny dinners daily.

The Buffalo Medical College admits women students, and two who took the junior course in pharmacy are sole proprietors and managers of a drug store in the city.

Canon Trench made All Saints' a free church seven years ago, the collection then amounting to 54,000 coins for the year. Now the collection is just double, so that in this case free seats have paid.

Burglars lately entered the house of a well-known surgeon and dentist in New Jersey, and after chloroforming the wife and daughter, went off with all the valuables. The chloroform killed the wife.

Mr. Austin Meldon, president of the Royal College of Surgeons in Ireland, has sent out invitations to meet her Excellency the Countess of Zetland at a conversazione to be held at the college on the 11th inst.

The Lord Mayor of Dublin has received a letter from Mr. Lees Knowles, M.P., secretary of the Guinness Trust, asking the co-operation of the Dublin Corporation in selecting sites for building dwellings for the poor.

Frederick Mills, sausage manufacturer, was fined £5 at Reading last week for having horseflesh on his premises without having the fact that he dealt in horseflesh for human food notified in conspicuous characters in front of the shop.

An anonymous donor has placed £1,000 in the hands of the Mayor of Cork, and £1,000 with the Rev. Canon Harley, rector of Christ Church, Cork, for the relief of poor widows in that city. The letters containing the gift bore the Cork postmark.

In twenty-eight of the largest English towns 6,450 births and 4,889 deaths were registered during the week ending January 4th. The annual rate of mortality in these towns, which had been 23·1, 23·5, and 21·8 per 1,000 in the preceding three weeks, rose last week to 25·2.

Margaret Barton, of Heysham, near Lancaster, died on Tuesday, after a few days' illness, at the reputed age of 104 years. She had to the last a remarkable memory and a fund of anecdotes; she had good eyesight, and up to a short time ago could read her Bible without spectacles.

The will of Joseph Schofield, of Boston, contains the following public bequests: Massachusetts Eye and Ear Infirmary, 2,500dols.; Perkins Institute for the Blind, 1,500dols.; Home for Aged Men, 1,000dols.; Kindergarten for the Blind, 3,000; Massachusetts General Hospital, 3,000dols.

A deputation of representatives from institutions for the education of deaf, dumb, and blind children waited on Dr. Craik, of the Education Department, at Edinburgh, on January 10th. Sheriff Guthrie Smith urged the peculiar claims of deaf, dumb, and blind children to a share in the benefits of the Education Act.

An outbreak of measles has occurred at Long Sutton, in the Spalding division of Lincolnshire. There are very few families in the parish who have not children suffering from the epidemic. A meeting of the School Board was held on Saturday, when it was decided to close the day and Sunday schools for a fortnight.

An unregistered letter, bearing the West London postmark, was delivered last week to Messrs. Mills, Bawtree and Co., bankers, Colchester. It contained a Bank of England note for £1,000 folded within a sheet of paper, on which was written, "For the Eastern Counties Idiot Asylum." There was no signature.

